# 2‐Pyrazoline‐5‐One Derivative Suppresses Cell Proliferation and Induces Apoptosis With Upregulation of Bax in Breast Cancer Cells

**DOI:** 10.1002/jbt.70975

**Published:** 2026-06-23

**Authors:** Sevgi Kocyigit Sevinc, Ş. Güniz Küçükgüzel, Sevim Rollas, Fulya Yukcu

**Affiliations:** ^1^ Faculty of Medicine, Biophysics Department Kütahya Health Sciences University Kütahya Turkey; ^2^ Faculty of Pharmacy, Pharmaceutical Chemistry Department Fenerbahçe University Istanbul Turkey; ^3^ Faculty of Pharmacy, Pharmaceutical Chemistry Department Marmara University Istanbul Turkey

**Keywords:** 2‐pyrazoline‐5‐one, apoptosis, BAX, breast cancer, cell migration, colony formation

## Abstract

The most common cancer in women worldwide is still breast cancer. Among its subtypes, luminal A and triple‐negative breast cancers present significant therapeutic challenges due to intrinsic drug resistance and absence of effective targeted treatments. The pharmacological potential of heterocyclic compounds has garnered more attention in recent years, and structures like pyrazolines, hydrazones, and triazoles are important in the development of new drugs. Compounds incorporating these scaffolds have been shown in numerous studies to have strong anticancer action. In this regard, pyrazoline derivatives have become a viable field of study for focused treatment approaches. This study focused on evaluating the cytotoxic potential of the 2‐pyrazoline‐5‐one derivative compound against luminal‐A (MCF‐7) and triple‐negative (MDA‐MB‐231) breast cancer cell lines. The CCK‐8 test was used to assess the impact on cell viability. Additionally, Annexin V/PI staining was used to identify the apoptotic effects, and fluorescence microscopy was used for analysis. The wound healing experiment was used to measure the capacity of cells to migrate, and migration process was monitored following scratch formation. The colony formation assay was used to assess the potential for cell proliferation, and clonal growth capacities were compared. Bax protein levels in compound‐treated cancer cells were quantified by ELISA to investigate the apoptotic mechanism. IC50 values in MCF‐7 and MDA‐MB‐231 cell lines were used to evaluate the cytotoxic effects of the 2‐pyrazoline‐5‐one derivative. The compound exhibited strong cytotoxic activity and reduced cell viability in both cell lines in a concentration‐dependent manner, with IC_50_ values of 13 µM in MCF‐7 cells and 16 µM in MDA‐MB‐231 cell. Furthermore, the compound effectively induced apoptosis and significantly suppressed colony formation and cell migration in both cell lines. Additionally, it showed a concentration‐dependent rise in Bax protein levels, suggesting a pro‐apoptotic action. These results confirm that the 2‐pyrazoline‐5‐one derivative has strong antiproliferative and pro‐apoptotic activity through Bax activation, providing a possible pathway for the creation of targeted treatments for breast cancer.

## Graphical abstract

2‐Pyrazoline‐5‐one derivative inhibits proliferation and migration and induces Bax‐mediated apoptosis in breast cancer cells.

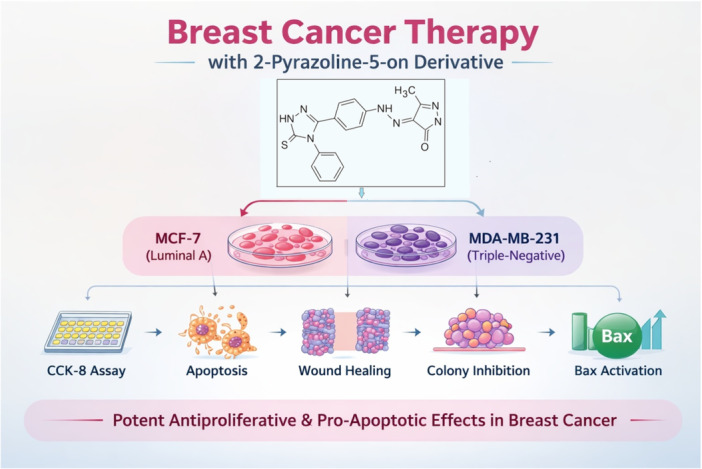

## 1 Introduction

Globally, women are more frequently diagnosed with breast cancer. It remains a major public health concern despite tremendous advancements in detection and treatment. Numerous risk factors, such as feminine sex, old age, genetic changes, and familial susceptibility, are linked to its development [1]. Because of the variability of its molecular makeup, breast cancer is divided into many subtypes. These include triple‐negative, HER2‐positive, luminal A, and luminal B, all of which have been thoroughly investigated for their unique traits. Because the luminal A and B subtypes include progesterone and estrogen receptors (ER/PR), they can be treated with hormone‐based medications. Triple‐negative breast cancer (TNBC), on the other hand, is linked to a more aggressive course of the disease because it lacks these receptors [2]. Despite the fact that luminal A breast cancer is typically associated with a favorable clinical result, there are still substantial treatment hurdles, especially because of acquired drug resistance. Similarly, the lack of effective targeted hormonal treatment options continues to make treating triple‐negative breast cancer difficult [3]. As a result, there is an urgent need to create new treatment approaches that can successfully target the triple‐negative and luminal A subtypes of breast cancer.

One of the most successful approaches in modern cancer research is the identification of substances that can cause cancer cell lines to undergo apoptosis and slow the growth of tumors. As a result, the creation of new chemotherapeutic drugs has been a major focus of current medicinal chemistry research. These agents aim to achieve enhanced efficacy and reduced toxicity compared to currently available treatments. In this context, heterocyclic compounds have gained considerable attention in drug development studies owing to their diverse pharmacological activities. In particular, compounds containing rings such as pyrimidine, triazole, hydrazide–hydrazone, oxadiazole, tetrazole, thiadiazole, and pyrazoline are among the most widely used scaffolds for novel drug discovery and the design of biologically active molecules. One of these heterocyclic rings, pyrazoline has attracted considerable attention and is widely employed in the synthesis of pharmaceutical active compounds. A wide range of pharmacological activity have been described for the 2‐pyrazoline ring, which has been well studied in the literature. These include anticancer, antimicrobial, monoamine oxidase inhibitory, antidepressant, neuroprotective, anticonvulsant, analgesic, anti‐inflammatory, antioxidant, antihypertensive, antihistaminic, and antiviral effects [4, 5, 6, 7]. Pyrazolines have attracted considerable attention due to their potential in preventing various types of cancer, including brain, bone, oral, oesophageal, gastric, hepatic, biliary, pancreatic, cervical, breast, prostate, lung, and colorectal cancers. Certain pyrazoline derivatives have also been identified as effective chemopreventive agents [6, 8, 9, 10]. Similarly, compounds containing triazole and hydrazone functional groups have also been widely reported in the literature to possess significant anticancer potential [11, 12].

One of the main ways that anticancer drugs work is by inducing apoptosis. In particular, regulating the survival and response to treatment of cancer cells depends on the mitochondrial (intrinsic) apoptotic pathway. The release of apoptotic agents from mitochondria into the cytoplasm is tightly regulated by members of the Bcl‐2 family of proteins, which are crucial regulators of the integrity of the mitochondrial outer membrane. Dysregulation of this pathway, which is often brought on by alterations in the expression or function of Bcl‐2 family proteins, promotes tumor growth and treatment resistance. The pro‐apoptotic protein BAX is one of these regulators that plays a crucial role in mitochondria‐mediated apoptosis. When BAX is activated, it causes mitochondrial outer membrane permeabilization (MOMP), which releases apoptogenic substances like cytochrome c, SMAC, and OMI. This causes caspase activation and cell death. However, anti‐apoptotic BCL‐2 family members either functionally suppress BAX or keep it in an inactive state in many cancer cells. Reduced sensitivity to chemotherapeutic drugs and resistance to apoptosis have been extensively linked to the overexpression of these anti‐apoptotic proteins. Because of this, direct BAX activation to encourage apoptosis has been suggested as a different therapeutic approach for the treatment of cancer [13].

As a result, direct pharmacological activation of BAX has become a viable substitute method for overcoming cancer cells' resistance to apoptosis. In this regard, it has been demonstrated that BTSA1, a small‐molecule BAX activator that has been pharmacologically optimized, binds specifically to the N‐terminal activation site of BAX, causes conformational changes, and encourages BAX‐dependent apoptosis. This substance has been demonstrated to display anticancer action in solid tumor cell lines such A549, HCT‐116, and PC‐3, diminish cell viability in a dose‐dependent way, and activate caspase‐3/7 in AML (human acute myeloid leukemia) and HeLa cells. Moreover, its novel analogue BTSA1.2 has demonstrated high cytotoxicity particularly in leukemia and lymphoma cells, showing a strong correlation with BAX expression levels [14, 15, 16, 17].

The many pharmacological effects of pyrazoline‐5‐one derivatives, especially their anticancer potential, have garnered significant interest in medicinal chemistry. In this context, the compound 3‐Methyl‐4‐[(2,4‐dihydro‐4‐phenyl‐1,2,4‐triazole‐3‐thione‐5‐yl)phenylhydrazono]−2‐pyrazoline‐5‐one used in this study represents a structurally distinct and multifunctional heterocyclic scaffold. It integrates 2‐pyrazoline‐5‐one, 1,2,4‐triazole‐3‐thione, and hydrazone functional groups within a single molecular framework [4]. The coexistence of these pharmacophores is particularly noteworthy, as both hydrazone and triazole derivatives have been extensively reported to exhibit significant anticancer activities, thereby supporting the pharmacological relevance of this compound. This supports the pharmacological relevance of this compound [8, 11, 12, 18].

Notably, this compound investigated in the present study has the same arylhydrazono‐2‐pyrazoline‐5‐one structural motif as BTSA1, which suggests that it may have BAX‐activating properties. This structural characteristic implies that the chemical may have antiproliferative and pro‐apoptotic effects on breast cancer cells, given the critical role that BAX‐mediated apoptosis plays in the killing of cancer cells.

Hence, we examined the effects of the 2‐pyrazoline‐5‐one derivative on key hallmarks of cancer, including cell proliferation, apoptosis induction, colony‐forming ability, and cell migration, using two breast cancer cell lines with distinct biological characteristics: the hormone receptor positive (Luminal‐A), less invasive MCF‐7 cell line and aggressive, metastatic MDA‐MB‐231 triple‐negative breast cancer (TNBC) cell line.

## 2 Materials and Methods

### 2.1 Synthesis of 3‐Methyl‐4‐[(2,4‐dihydro‐4‐phenyl‐1,2,4‐triazole‐3‐thione‐5‐yl)phenylhydrazono]−2‐pyrazoline‐5‐one and Cellular Application

The 2‐pyrazoline‐5‐one derivative was synthesized, purified, and structurally characterized in this study using the procedure outlined by Küçükgüzel ŞG et al. (2000). After then, 30 mM stock solution was made by dissolving it in 100% dimethyl sulfoxide (DMSO). For cell treatment, intermediate working solutions were prepared from the stock solution using DMEM medium. MCF‐7 and MDA‐MB‐231 breast cancer cells were treated with the compound at 10, 25, and 50 μM. Following a 24 h treatment period, the cells were collected and ready for further examination.

### 2.2 Cell Culture

Dulbecco's Modified Eagle Medium (DMEM) supplemented with 10% fetal bovine serum (FBS) and 1% penicillin‐streptomycin was used to sustain MCF‐7 and MDA‐MB‐231 cells. The culture media was changed three times a week while the cells were cultivated at 37°C in a humidified environment with 5% CO_2_.

### 2.3 Cell Viability and Cytotoxicity

The 2‐pyrazoline‐5‐one derivative's effects on cell survival and cytotoxicity were assessed in vitro using the CCK‐8 colorimetric test. After seeding 5 × 10^3^ cells per well into 96‐well plates, the cells were left to adhere for a full day. Cells were then exposed to escalating doses of the compound (10, 25, and 50 μM) for a further 24 h. Untreated cells and cells treated with 0.1% DMSO made up the control groups. After the treatment period, 100 μL of new medium containing 10 μL of CCK‐8 reagent (Cell Counting Kit‐8, BMU106‐EN, Abbkine) was added to the culture medium. A microplate reader was used to measure absorbance at 450 nm following a 4 h incubation period at 37°C. Every experiment was run in triplicate (*n* = 3). Cell viability did not differ statistically significantly between the control groups with or without 0.1% DMSO. GraphPad Prism version 6.01 was used to calculate the half‐maximal inhibitory concentration (IC_50_) values.

### 2.4 Annexin‐V/Propidium Iodide Staining for Apoptosis Detection

MCF‐7 and MDA‐MB‐231 cells were seeded at a density of 4 × 10^2^ cells per well into 24‐well plates with sterile round coverslips for apoptosis measurement. The cells were then incubated for 24 h at 37°C in a humidified environment with 5% CO_2_. The 2‐pyrazoline‐5‐one derivative was then applied to the cells for 24 h at the specified doses. After treatment, cells were stained with 10 μL FITC‐Annexin V and 2 μL PI in the dark for 30 min after being rinsed with 250 μL of 1× Annexin binding solution (FITC Annexin V Apoptosis Detection Kit, ABPBİO A026). Coverslips were mounted onto microscope slides and then apoptotic and necrotic cells were visualised under a fluorescence microscope.

### 2.5 Colony Formation Test

Six‐well plates were seeded with MCF‐7 (1000 cells/well) and MDA‐MB‐231 (500 cells/well) cells. The next day, the plates were treated with the 2‐pyrazoline‐5‐one derivative at concentrations between 0 and 50 µM for 24 h. Following treatment, MCF‐7 cells were incubated for 2 weeks and MDA‐MB‐231 cells for 3 weeks at 37°C in a humidified environment with 5% CO2. The medium was changed every 2 days. Following the incubation time, cells were fixed with 4% paraformaldehyde for 20 min, rinsed with PBS, then stained for 30 min at room temperature with 0.5% crystal violet. ImageJ software was used to examine the quantity and area of colonies with at least 50 cells.

### 2.6 Cell Migration for Wound Healing (Scratch) Assay

The migratory capacity of cells was assessed using an in vitro wound healing test. In 6‐well plates, MCF‐7 and MDA‐MB‐231 cells were planted and cultivated until they achieved 80%–90% confluence. A 20 µL pipette tip was used to make a linear scratch in each well, and detached cells were gently washed with 1X PBS. A light microscope was used to take the first pictures at 0 h. To minimize cell proliferation, control and treated wells received medium containing 1% FBS. Wound areas were reimaged with a microscope following a 24 h incubation period with the 2‐pyrazoline‐5‐one derivative. ImageJ was used to measure the scratch areas at 0 and 24 h, and the wound width was calculated.

### 2.7 Bax Protein Analysis

The total protein of MCF‐7 and MDA‐MB‐231 cells was measured after they were exposed to 0–50 μM of the 2‐pyrazoline‐5‐one derivative for a whole day. Bax levels were determined using a Human Bax ELISA Kit (A.B.T, ABT1315Hu). Standards were prepared by serial dilution from a 50 ng/mL stock. Microplate wells were filled with 100 µL of standards, blanks, and samples, and they were incubated for 90 min at 37°C. Following washing, 100 µL of biotinylated detection antibody was added and allowed to incubate for 60 min. Next, 100 µL of HRP conjugate was added and allowed to incubate for 30 min. After cleaning the wells and adding 100 µL of substrate solution, the reaction was halted with 50 µL of stop solution after 15 min in the dark. After measuring absorbance at 450 nm, Bax protein levels were computed using the standard curve and normalized to total protein.

### 2.8 Statistical Analysis

Statistical analyses were performed using GraphPad Prism version 6.01 (GraphPad Software, CA, USA). The Student's t‐test was used to evaluate the data. The results are displayed as mean ± SD. *p < 0.05, **p < 0.01, ***p < 0.001, and ****p < 0.0001 were the thresholds for statistical significance.

## 3 Results

### 3.1 Chemistry

**Figure 1:**
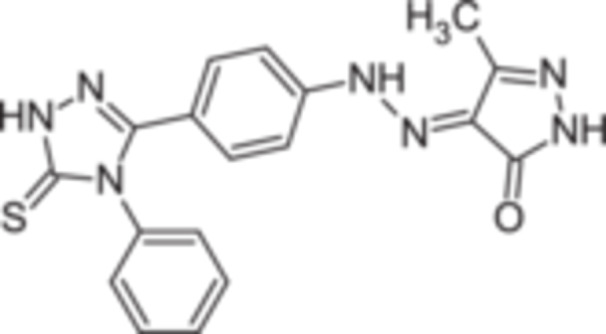
3‐Methyl‐4‐[(2,4‐dihydro‐4‐phenyl‐1,2,4‐triazole‐3‐thione‐5‐yl)phenylhydrazono]−2‐pyrazoline‐5‐one.

The 2‐pyrazoline‐5‐one derivative, namely 3‐methyl‐4‐[(2,4‐dihydro‐4‐phenyl‐1,2,4‐triazol‐3‐thion‐5‐yl) phenylhydrazono]−2‐pyrazoline‐5‐one (Figure 1), was synthesized using the procedures previously described by Küçükgüzel and colleagues [4]. Its purity was verified by thin‐layer chromatography (TLC) and microanalysis (CHNS), and its structure was characterised using FT‐IR, ^1^H NMR, ^13^C NMR, and EI‐mass spectroscopy.

### 3.2 Cytotoxic and Antiproliferative Effects of 2‐Pyrazoline‐5‐One Derivative

**Figure 2:**
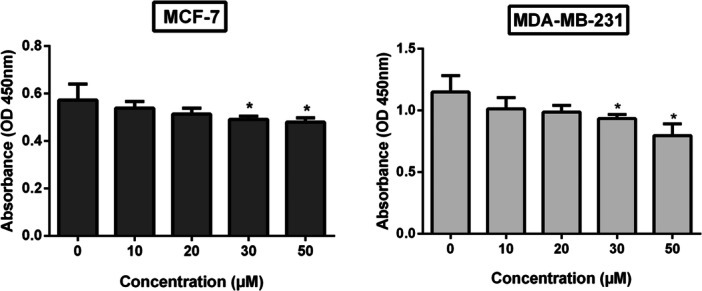
The cell viability of both MCF‐7 and MDA‐MB‐231 cells was assessed by a CCK‐8 assay after 24 h with increasing compound concentrations. Data from three separate trials (*n* = 3) are shown as mean ± SD, and statistical significance in comparison to the control is highlighted (* *p* < 0.05).

The cytotoxic effect of the 2‐pyrazoline‐5‐one derivative was evaluated in MCF‐7 and MDA‐MB‐231 cells after a 24 h treatment period at various concentrations (0–50 µM), and cell survival was determined through the CCK‐8 method. The compound markedly reduced cell viability in different cancer cell lines in a concentration‐dependent way (Figure 2). In MCF‐7 cells, optical density readings gradually decreased with increasing concentrations, with statistically significant reductions particularly at 30 and 50 µM. MDA‐MB‐231 cells showed a similar concentration‐dependent drop in cell proliferation. Based on these analyses, the IC_50_ (50% inhibitory concentration) values of the compound were calculated as 13 µM in MCF‐7 cells and 16 µM in MDA‐MB‐231 cells.

**Figure 3:**
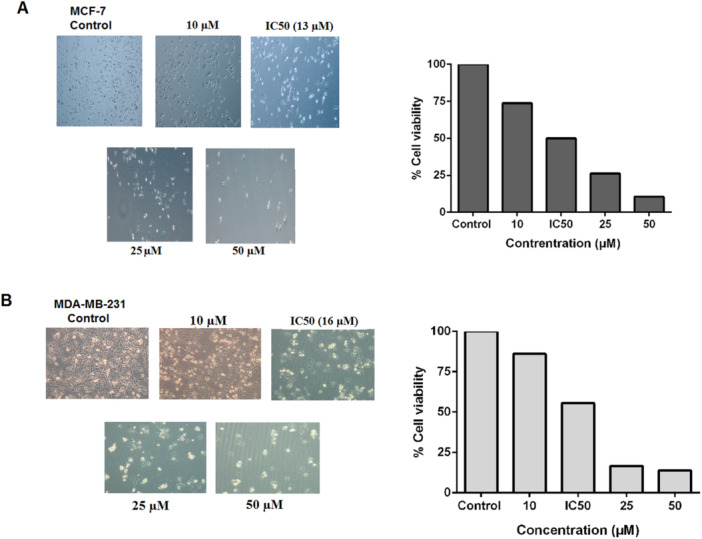
Trypan blue exclusion assay findings and typical light microscopy pictures demonstrate a dose‐dependent decrease in the viability in MCF‐7 (A) and MDA‐MB‐231 (B) cells after 24 h of treatment with the compound (10–50 µM and IC_50_).

Light microscopy and the Trypan Blue exclusion experiment in breast cancer cells confirmed the compound's anti‐proliferative action (Figure 3). The cytotoxicity of the compound was evaluated in MCF‐7 and MDA‐MB‐231 cells through morphological assessment and viability analysis Figure 3A,B).

In MCF‐7 cells (Figure 3A), compound treatment induced dose‐dependent morphological alterations, including decreased cell density, cell rounding, detachment, and increased cellular debris compared with control cells. Cell viability decreased to 50% at the IC_50_ concentration. Similarly, MDA‐MB‐231 cells (Figure 3B) exhibited pronounced dose‐dependent cytotoxic effects, characterized by cell shrinkage, rounding, detachment, and reduced cell numbers relative to controls. Cell viability decreased to 55% as a result of the IC_50_ concentration.

Overall, these findings indicate that the compound exerts a significant, dose‐dependent cytotoxic effect in each of the cell lines.

### 3.3 Apoptotic Effect of 2‐Pyrazoline‐5‐One Derivative in Breast Cancer Cells

**Figure 4:**
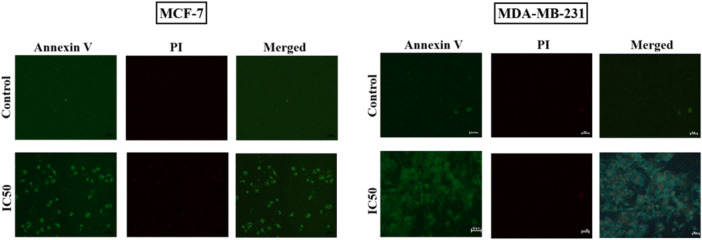
The presentation of fluorescence microscopy images of MCF‐7 and MDA‐MB‐231 breast cancer cells in control and IC_50_‐treated groups after they were incubated with the 2‐pyrazoline‐5‐one derivative for 24 h and then stained with Annexin V/PI (*n* = 3).

Using the Annexin V/PI double‐staining approach, the apoptotic effects of the 2‐pyrazoline‐5‐one derivative were assessed in MCF‐7 and MDA‐MB‐231 cells. The two cell lines in the control groups showed very little Annexin V and PI positivity, suggesting that the great majority of cells were still viable. On the other hand, groups exposed to the compound at the IC_50_ concentration showed a significant raised the number of Annexin V‐positive cells (Figure 4).

In MCF‐7 cells, IC_50_ treatment caused a pronounced rise, particularly in early apoptotic cells, which are characterized by an Annexin V‐positive and PI‐negative population. Merged images clearly demonstrated an enhanced density of apoptotic cells. Likewise, in MDA‐MB‐231 cells, a significant increase in Annexin V positivity was detected following IC_50_ treatment, and merged images showed significantly more apoptotic cells than the control group. These findings indicate that the 2‐pyrazoline‐5‐one derivative effectively induces apoptotic process in two different cancer cells and exerts its antiproliferative effect primarily by activating programmed cell death.

### 3.4 Inhibitory Effect of 2‐Pyrazoline‐5‐One Derivative on Colony Formation

**Figure 5:**
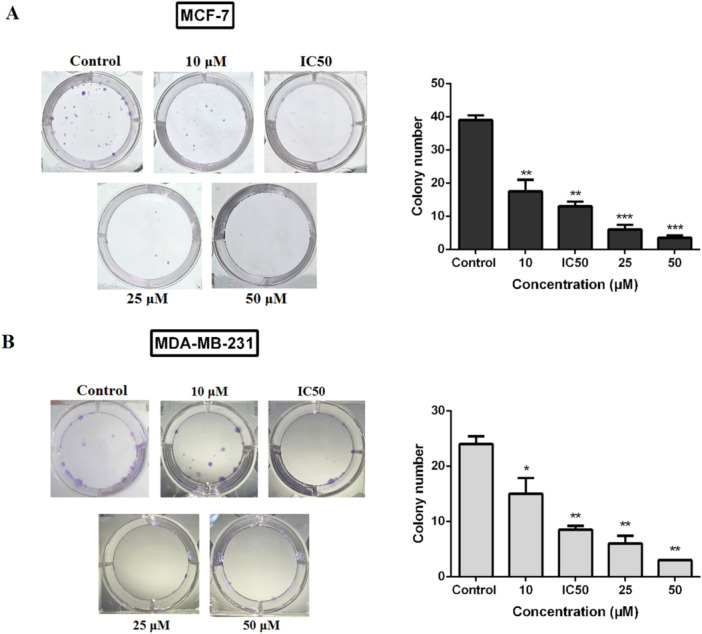
Clonogenic assay demonstrating the inhibitory effect of the 2‐pyrazoline‐5‐one derivative on long‐term survival of MCF‐7 (A) and MDA‐MB‐231 (B) cancer cells. Cells were cultured for 14–21 days (*n* = 2) following a 24 h treatment. * *p* < 0.05, ** *p* < 0.01, and *** *p* < 0.001 with regard to the control.

The colony formation assay demonstrated that treatment with the 2‐pyrazoline‐5‐one derivative significantly suppressed clonogenic survival in both breast cancer cells in a concentration‐related way (Figure 5). Representative images clearly show a progressive reduction in colony number with increasing concentrations of the compound.

In MCF‐7 cells, colony numbers decreased markedly starting at 10 µM and were significantly reduced at IC_50_ and higher concentrations. At 50 µM, colony formation was nearly completely inhibited. Similarly, MDA‐MB‐231 cells displayed a notable dose‐dependent decline in colonial numbers, with substantial suppression observed at IC_50_ and above, and minimal colony formation at 50 µM.

Overall, these findings suggest that the compound effectively inhibits a longer‐term proliferation potential and clonogenic survival of two different cancer cells, with a more pronounced suppressive effect at higher concentrations.

### 3.5 Cell Migration Analysis by Wound Healing (Scratch) Assay

**Figure 6:**
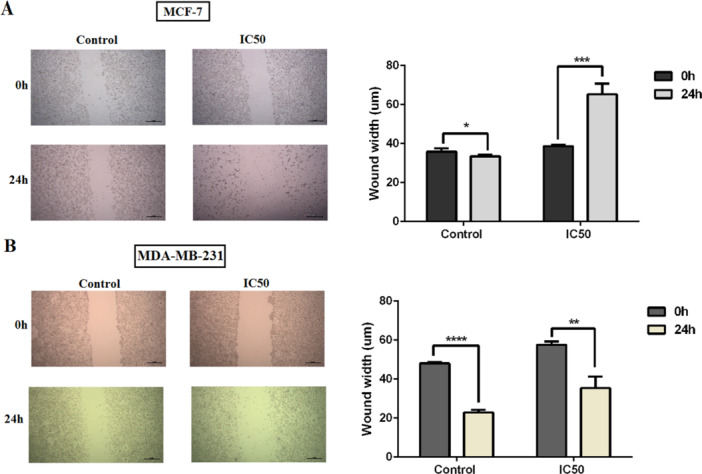
A wound healing test was used to evaluate the impact of IC_50_ treatment on cell migration in MCF‐7 (A) and MDA‐MB‐231 (B) cells. The related quantification of wound width (µm) and representative images for 0 and 24 h are displayed. IC_50_ treatment significantly inhibited wound closure after 24 h (*n* = 3). * *p* < 0.05, ** *p* < 0.01, *** *p* < 0.001, and **** *p* < 0.0001.

2‐pyrazoline‐5‐one derivative significantly inhibited cell migration in both breast cancer cells. After 24 h, partial wound closure was observed in the control group of the MCF‐7 cells. However, compound treatment with IC_50_ concentration resulted in a significant enhancement of wound width against the control group. Quantitative analysis confirmed that wound closure was significantly suppressed in IC_50_‐treated group (*p* < 0.001), indicating strong inhibition of migratory capacity (Figure 6A).

Similarly, MDA‐MB‐231 cells displayed substantial wound closure in the control group at 24 h. However, treatment with IC_50_ concentration significantly reduced wound closure (*p* < 0.01) at 24 h (Figure 6B). Although MDA‐MB‐231 cells possess higher intrinsic migratory potential, the compound effectively attenuated their migration.

### 3.6 Effect of Treatment on Bax Protein Expression in Breast Cancer Cells

**Figure 7:**
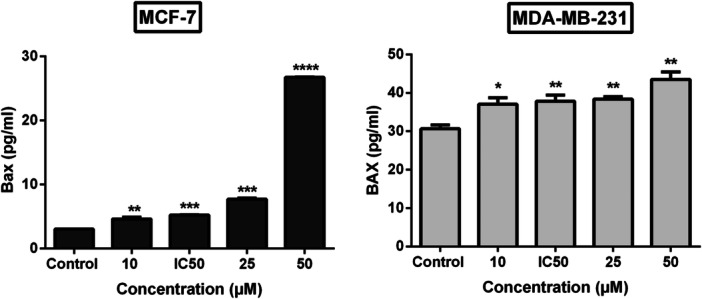
Effect of the 2‐pyrazoline‐5‐one derivative on Bax protein levels in MCF‐7 and MDA‐MB‐231 cells after a 24 h exposure (*n *= 3). * *p* < 0.05, ** *p* < 0.01, *** *p* < 0.001, and **** *p* < 0.0001 versus to the control.

The ELISA approach was used to assess changes in Bax protein levels in the breast cancer cell lines MCF‐7 and MDA‐MB‐231. In comparison to the control group, Bax levels increased in two types of cell at all tested concentrations of the 2‐pyrazoline‐5‐one derivative (Figure 7).

Bax expression in MCF‐7 cells increased markedly in a concentration‐dependent manner, particularly at IC_50_, 25 µM, and 50 µM, this increase was proved to be extremely and very highly statistically significant (*p* < 0.001; *p* < 0.0001). At the IC_50_ concentration, Bax protein levels increased by 72% in a comparison with the control group.

In MDA‐MB‐231 cells, the increase in Bax levels was more moderate; however, it became statistically significant starting from 10 µM (*p* < 0.05) and showed a consistent upward trend while maintaining significance at IC_50_, 25 µM, and 50 µM (*p* < 0.01). At the IC_50_ concentration, Bax levels increased by 23% relative to the control group, indicating a meaningful and consistent apoptotic response to the compound in MDA‐MB‐231 type cells as well.

These findings show that 2‐pyrazoline‐5‐one derivative exerts pro‐apoptotic effects in two types of cells; however, MCF‐7 cells exhibit a stronger Bax‐mediated apoptotic response than MDA‐MB‐231 cells. Overall, the outcomes suggest that the compound triggers apoptosis with upregulating BAX expression. As a result, this compound may cause cancer cells to undergo apoptosis by activating BAX. It could potentially act as a BAX activator, similar to BTSA1, in terms of its mechanism of action.

## 4 Discussion

Incidence of cancer is expected to increase significantly over the next several decades, making it the second most common cause of death globally, after cardiovascular disorders [19]. Breast cancer is the most common cancer diagnosed in women and a major contributor to cancer‐related mortality. Its molecular heterogeneity—particularly the distinction between a typical hormone receptor–positive (luminal) and triple‐negative subtypes—continues to present significant therapeutic challenges [20]. The development of novel agents capable of selectively inducing apoptosis while suppressing tumor progression remains a major focus in translational oncology.

In the present study, we demonstrate that a multifunctional 2‐pyrazoline‐5‐one derivative exerts potent anticancer activity in both luminal A (MCF‐7) and triple‐negative (MDA‐MB‐231) breast cancer cell lines. Notably, the compound displayed low micromolar IC_50_ values (13 µM for MCF‐7 and 16 µM for MDA‐MB‐231), indicating strong cytotoxic efficacy across molecularly distinct subtypes. The slightly greater sensitivity observed in MCF‐7 cells suggests that hormone receptor–positive breast cancer cells may be more susceptible to this scaffold. This observation is consistent with previous reports describing enhanced antiproliferative activity of pyrazoline derivatives in ER‐positive models [21, 22].

Importantly, the cytotoxicity observed was accompanied by classical apoptotic morphology and a marked increase in Annexin V‐positive populations, indicating that apoptosis—rather than nonspecific necrosis—constitutes the primary mechanism of cell death. Such findings are consistent with past research showing that pyrazoline‐ and hydrazone‐based heterocycles promote apoptosis [23, 24]. The predominance of early apoptotic cells at IC_50_ concentrations suggests that the compound initiates programmed cell death efficiently and at an early stage of treatment.

Beyond short‐term cytotoxicity, the compound significantly suppressed clonogenic survival in both cell lines. Since clonogenic assays reflect capacity of individual tumor cells to undergo unlimited division and contribute to recurrence, this observation is particularly relevant. The marked inhibition of colony formation at IC_50_ and higher concentrations indicates durable antiproliferative activity and suggests that the compound may interfere with long‐term tumor sustainability. Similar sustained suppression of clonogenic potential has been reported for hybrid heterocyclic anticancer agents integrating multiple pharmacophores [25].

The therapeutic importance of these discoveries is further reinforced by the suppression of cell migration. Increased invasiveness and metastatic potential are characteristics of triple‐negative breast cancer. The observation that migration was significantly attenuated even in MDA‐MB‐231 cells underscores the compound's potential antimetastatic activity. Similar effects have been reported in previous studies showing that triazole‐ and pyrazoline‐based heterocyclic compounds can impair migration [26, 27] and related malignant phenotypes in breast‐derived cancer cells.

Mechanistically, the study's most striking outcome is the significant upregulation of BAX protein levels following treatment. BAX is a central effector of mitochondrial outer membrane permeabilization, the irreversible step that commits cells to intrinsic apoptosis [28]. The observed dose‐dependent increase in Bax expression—particularly 72% elevation in MCF‐7 cells at IC_50_—strongly implicates activation of the mitochondrial apoptotic pathway. The comparatively attenuated increase in MDA‐MB‐231 cells (23%) may reflect the intrinsically higher apoptotic threshold and anti‐apoptotic protein dominance in TNBC cells.

The structural relevance of this finding is noteworthy. The tested compound shares the arylhydrazono‐2‐pyrazoline‐5‐one scaffold with BTSA1. BTSA1 is a direct BAX activator that has been pharmacologically optimized. It interacts with BAX's N‐terminal activation site and causes conformational activation [13]. BTSA1 and its derivatives have demonstrated pro‐apoptotic activity in hematologic and solid tumor models, typically within the low‐to‐mid micromolar range [16, 17]. The IC_50_ values observed in our study suggest that this compound may exhibit a similar functional profile.

While our current data demonstrate increased BAX expression, it is possible that the compound may facilitate the conformational activation of BAX or interfere with its inhibitory interactions with members of the anti‐apoptotic BCL‐2 family. If confirmed, this dual capacity to upregulate and activate BAX would establish the molecule as a mechanistically targeted modulator of apoptosis. As resistance to apoptosis is a characteristic of aggressive breast cancers, the pharmacological reactivation of BAX is a highly attractive therapeutic strategy.

To our knowledge, direct BAX‐targeting small molecules have not been extensively evaluated in parallel across luminal and triple‐negative breast cancer models. Therefore, the present findings provide novel insight into BAX‐mediated apoptotic modulation in distinct breast cancer subtypes and highlight the translational potential of arylhydrazono‐pyrazolinone scaffolds in solid tumors.

## 5 Conclusion

In conclusion, the 2‐pyrazoline‐5‐one derivative demonstrates pronounced anticancer activity in both luminal A (MCF‐7) and triple‐negative breast cancer (MDA‐MB‐231) cells. Our compound induces dose‐dependent cytotoxicity, promotes apoptosis, suppresses clonogenic survival, inhibits migration, and significantly upregulates Bax expression. The marked increase in Bax levels suggests the engagement of the intrinsic apoptotic pathway, indicating the activation of mitochondria‐mediated apoptotic signaling. This observation raises the possibility that this molecule may function as a Bax‐modulating or Bax‐activating agent. The present findings highlight this scaffold as a potential candidate for mitochondria‐targeted therapeutic strategies in breast cancer.

## Author Contributions

**Sevgi Kocyigit Sevinc:** conceptualization, investigation, funding acquisition, writing – original draft, methodology, validation, visualization, writing – review and editing, project administration, supervision, resources. **Ş. Güniz Küçükgüzel:** writing – original draft, methodology, investigation. **Sevim Rollas:** methodology, investigation. **Fulya Yukcu:** methodology.

## Ethics Statement

Since this work used commercially available, established cell lines, ethical approval was not necessary. Research involving human or animal subjects that needs ethics committee approval does not use these cell lines.

## Consent

The authors have nothing to report.

## Conflicts of interest

The authors declare no conflicts of interest.

## Data Availability

The corresponding author can provide the data supporting the findings of this study upon reasonable request. The data supporting the conclusions of this article are included within the article.
